# Cancer screening outside of age recommendations: a population-based study

**DOI:** 10.1186/s12889-025-22848-4

**Published:** 2025-05-06

**Authors:** Frerik Smit, Vladimir Jolidon, Bernadette WA van der Linden, Nicolas Rodondi, Stéphane Cullati, Arnaud Chiolero

**Affiliations:** 1 Population Health Laboratory (#PopHealthLab), University of Fribourg, Fribourg, Switzerland; 2 Swiss School of Public Health (SSPH+), Zurich, Switzerland; 3 Institute for Primary Health Care (BIHAM), University of Bern, Bern, Switzerland; 4 Department of General Internal Medicine, Inselspital, Bern University Hospital, University of Bern, Bern, Switzerland; 5 School of Population and Global Health, McGill University, Montreal, Canada

**Keywords:** Cancer screening, Low-value care, Evidence-based medicine, Older adults, Cancer prevention

## Abstract

**Background:**

Cancer screening outside of evidence-based recommendations can be considered a form of low-value care. We aimed to describe the frequency of colorectal, breast, cervical, and prostate cancer screening outside of recommended age guidelines in Switzerland.

**Methods:**

We analysed data from the 2022 Swiss Health Survey. Of 21,930 participants aged 15 or more, 20,515 (9,555 men and 10,960 women) were included in this study. We calculated age at last screening and classified individuals as having been not screened, screened within age-specific A, B, and C recommendations from the United States Preventive Services Taskforce (USPSTF), screened within age-specific A and B recommendations, or screened outside of recommendations.

**Results:**

Among adults aged 75 years and above (75+), 40.2% (men: 35.1%; women: 44.5%) had undergone cancer screening outside of USPSTF A, B, and C recommendations. This proportion was 26.0% for adults aged 85+ (men: 27.8%; women: 24.6%). Cervical cancer screening was the most frequently undertaken outside of recommended ages by older adults (women aged 75+: 37.1%), followed by prostate (men aged 75+: 34.0%), breast (women aged 75+: 17.8%), and colorectal cancer screening (adults aged 75+: 1.3%). Screening outside of recommendations was also observed among middle-aged adults 40–59 at 12.3% (men: 20.8%; women 4.0%), and younger-aged women 20–39 at 9.9%. Proportions for screening outside of USPSTF A and B recommendations were high (adults 75+: 50.4%; adults 85+: 40.6%; adults 40–59: 20.9%).

**Conclusions:**

Cancer screening outside of recommendations is highly prevalent, particularly among older adults. Further research is needed to better understand drivers of this form of low-value care.

**Supplementary Information:**

The online version contains supplementary material available at 10.1186/s12889-025-22848-4.

## Introduction

A core principle of cancer screening is that only screening whose benefits outweigh its harms at a reasonable cost should be recommended [1–3]. High-quality evidence on the benefits and harms of specific types of screening is, therefore, critical for ensuring that cancer screening is beneficial to population health [1]. Well-conducted trials along with systematic reviews and meta-analyses are particularly important sources of evidence that enable independent expert bodies to develop cancer screening guideline recommendations – notably such as those from the United States Preventive Services Task Force (USPSTF) [4] – and that provide a critical decision-making tool for healthcare practitioners and policymakers in guiding cancer screening practices [1, 4, 5]. The quality of evidence, accounting for the internal and external validity of screening studies, is a major criterion in the drafting of recommendations [4]. In turn, screening occurring outside of these recommendations can be considered a form of low-value care [3], where existing evidence does not show that it provides a meaningful benefit, benefits are not outweighed by harms, or benefits are insufficient proportional to costs [6].

A principal component of recommendations is the age groups targeted for undergoing specific types of cancer screening. This includes the age at which cancer screening should be initiated, as well as the age for screening cessation [7–10]. Correspondingly, people who undergo cancer screening outside of recommended ages are exposed to the potential harms of screening without evidence suggesting that they can experience a meaningful benefit. Notably, studies comprehensively assessing the frequency of cancer screening outside of recommended ages have predominantly been undertaken in the United States and Canada [11–17]. One study conducted in the United States found that screening above USPSTF age recommendations occurred among 45.8–74.1% of community-dwelling older adults depending on screening type [11]. In Canada, a study showed that 24% of adults 40–49 years of age had been screened for cancer despite the absence of recommendation for screening before the age of 50 years [15]. Further, when looking at the European context, two studies from France showed that mammography screening outside of recommendations is frequent among women aged below 50 and above 75 at 78% and 51%, respectively [18, 19]. Cancer screening outside of recommendations is, therefore, likely frequent in other high-income countries, which can benefit from a detailed analysis of screening practices by age and recommendation grades to inform policy toward the prevention of low-value care. Our aim was therefore to describe the frequency of colorectal, breast, cervical, and prostate cancer screening outside of recommended age guidelines in Switzerland.

## Methods

### Data source

This study – adherent to the strengthening the reporting of observational studies in epidemiology (STROBE) checklist [20] (Table S7) – is a secondary analysis of the of the 2022 Swiss Health Survey [21], which is a large population-based cross-sectional survey designed to assess heath and health behaviours of the Swiss population undertaken every five years by the Swiss Federal Office of Statistics since 1992. This survey, which is carried out in accordance with Swiss federal law, allows residents of Switzerland from the age of 15 to be recruited for participation in the survey without needing to obtain formal written consent. Nevertheless, all invited participants were provided with relevant information about the Swiss Health Survey and were given the option of declining and/or withdrawing participation. A total of 60,651 Swiss residents aged 15+ were invited to participate following randomised multistage probability sampling, of which 21,930 people participated (36.2% participation rate; Figure S1).

### Population

The target population we aim to generalise our results to is the population of Switzerland [22]. In turn, the sample under study (*N* = 20,515) comprised of all participants of the 2022 Swiss Heath Survey after excluding participants with missing data on cancer screening (*N* = 729) and proxy respondents (*N* = 686) – who are close persons responding on behalf of participants incapable of answering questions themselves – as they were not posed screening-related questions (Figure S1).

### Variables of interest

Participants underwent computer-assisted telephone interviews and were also invited to complete a written questionnaire, which can be found elsewhere [23]. Most data are self-reported by participants, with certain variables being ascertained from cantonal registries. Our outcomes of interest were screening use for four types of cancer, namely, colorectal, breast, cervical, and prostate cancer. All participants aged 40+ were asked about screening for colorectal cancer (faecal occult blood test (FOBT) and/or colonoscopy), women aged 20+ were asked about breast and cervical cancer screening, and men aged 40+ were asked about screening for prostate cancer. Participants were asked whether they underwent a specific detection test, when they last underwent that test (colonoscopy: less than 12 months, 1 to less than 5 years ago, 5 to less than 10 years ago, or 10+ years ago; other screening types: less than 12 months, 1 to less than 2 years ago, 2 to less than 3 years ago, 3 to less than 5 years ago, or 5+ years ago), and the reason for that last test. More detailed information on the ascertainment methods of these variables and of secondary variables of interest are provided in the supplementary material (pp.3–4).

### Swiss healthcare system

Federal law requires all residents of Switzerland to have a mandatory basic health insurance coverage, where individuals can select health insurance plans with varying deductibles. After an individual reaches their deductible limit, healthcare costs become subject to a capped 10% copay. Opportunistic screening for common cancer screening types is generally reimbursed by basic insurance coverage but is subject to the deductible and copay. However, in cantons (states) where organised breast cancer or colorectal cancer screening programs exist, screening that occurs within said programs are typically exempt from the deductible but subject to the copay [24].

### Referent screening guidelines

We defined screening within and outside of recommendations for specific screening types and age groups according to USPSTF recommendations released up to 2022, the year the survey was conducted. Notably, given that USPSTF guidelines are based on the synthesis of studies conducted both within and outside of the United States, their recommendations are not exclusively applicable to the United States context and have the potential to be justifiably used as referent guidelines in other countries as well [7–10]. In the context of Switzerland, there is no singular authoritative body that provides cancer screening recommendations and USPSTF guidelines are considered a valid source of recommendations that are frequently referred to by Swiss healthcare providers and policymakers [25]. Further, certain organisations in Switzerland – such as EviPrev – have produced recommendations that are explicitly based on the USPSTF recommendations [25].

Screening recommendations from the USPSTF are provided with accompanying grades that correspond to the certainty and magnitude of net benefit of specific screening modalities in specific populations: A (high certainty of substantial net benefit), B (high or moderate certainty of moderate net benefit), C (at least moderate certainty of small net benefit), D (high or moderate certainty of net null benefit or net harm), and I (insufficient existing evidence to make an assessment) [4]. We reported cancer screening use according to two sets of recommendations, that is, A, B, and C recommendations and A and B recommendations, respectively. The first set of recommendations encompasses all screening for which evidence shows a net benefit – irrespective of its magnitude – and the second set of recommendations only encompasses screening for which evidence indicates that the net benefit is moderate to substantial. Correspondingly, the USPSTF suggests that only recommendations with an A or B grade should be offered to all individuals in specified groups, while recommendations with a C grade should be offered to select individuals in specified groups following individualised decision-making [4]. Reporting results separately for these two sets of recommendations thereby allowed us to capture how screening use differs according to recommendations with varying levels of strength of evidence and net benefit.

As summarised in Figure S2, for colorectal cancer, the 2021 guidelines recommend screening to adults aged 50–75 (A grade), adults 45–49 (B grade), and adults 76–85 (C grade) [8]. For breast cancer, the 2016 guidelines recommend screening to women 50–74 (B grade) and 40–49 (C grade) years old [7]. For cervical cancer, 2018 guidelines recommend screening to women 21 to 65 years of age (A grade) [9]. For prostate cancer, the 2018 USPSTF guidelines recommend screening to men 55–69 years old (C grade) [10].

### Statistical analysis

We estimated the frequency of cancer screening according to USPSTF age-specific recommendations in four steps. First, we calculated age at screening of individuals who underwent specific testing modalities explicitly for preventive purposes by subtracting the assumed time of screening from a participant’s age. More specifically, assumed time of screening for adults less than 60 years old was the lower bound of the self-reported time of last test (e.g., adults aged less than 60 who screened “2 years to less than 3 years ago” were assumed to have been screened exactly 2 years ago). For adults 60+ years old, assumed time of screening was the upper bound of the self-reported time of last test (e.g., adults aged 60+ who screened “2 years to less than 3 years ago” were assumed to have been screened exactly 3 years ago). Adults 60+ years old who screened 5+ years ago (10+ for colonoscopy) were assumed to have been screened within recommendations.

Second, for each modality, participants were classified as having been not screened (which includes individuals who have undergone the test, but their last test was for non-preventive purposes), screened within USPSTF age-specific recommendations (A, B, and C recommendations and A and B recommendations separately), or screened outside of recommendations. While these calculations result in underestimates of frequency of screening outside of recommendations, this method ensures that participants are not misclassified as having undergone non-recommended screening. Third, we also undertook calculations for any cancer screening, where participants who had never undergone any type of screening or only undergone diagnostic tests were classified as not screened, participants who had undergone screening tests which all occurred at recommended ages were classified as having been screened within recommendations, and participants who had undergone screening that included at least one screening test outside of recommended ages were classified as having been screened outside of recommendations. Similar calculations were undertaken for any colorectal cancer screening.

Fourth, reported proportions were weighted using survey sampling weights calculated by the Office of Federal Statistics, which partially correct for survey non-participation and increase the national representativeness of results [26] based on region, sex, age, nationality, civil status, education, language, revenue, and household size and type.

### Estimating misclassification of age at screening

Our method for calculating age at screening results in underestimates of the proportion of participants who have been screened outside of given age recommendations. This underestimate is likely to be particularly large for adults 60+ years of age who responded having been screened 5+ years ago (10+ years ago for colonoscopy), as they were all classified as having been screened within recommendations. However, whereas participants of the 2022 survey were asked to indicate the time ranges in which they last underwent a screening test, participants of the 2017 survey were asked to provide the specific year they last underwent specific screening tests. Therefore, using this data from the 2017 survey, we estimated the proportion of participants 60+ years of age who underwent screening 5+ years ago (10+ for colonoscopy) that were misclassified as having been screened within age recommendations in the 2022 survey. Further details are provided in the supplementary material (pp.6–7).

## Results

Weighted proportions of the characteristics of the included 20,515 participants (9,555 men and 10,960 women) of the 2022 Swiss Health Survey are reported in Table 1. In terms of socio-demographic characteristics, 14.1% were 75 years old or above, 85.8% had either a secondary or tertiary degree, 76.9% were Swiss citizens, and 61.8% lived in urban settings. For health-related characteristics and behaviours, 85.9% of participants self-reported either good or very good health, 64.6% did not report having a chronic condition or long-term health issue, 82.7% had engaged in any medical consultation in the past year, and 74.9% were physically active on a weekly basis.

**Table 1:** Characteristics of included 2022 Swiss Health Survey participants (weighted proportions)

	All	Men	Women
	(N = 20,515)	(N = 9,555)	(N = 10,960)
**Socio-demographic characteristics**			
Age groups			
85+	2.30%	2.00%	2.50%
75–84	11.80%	11.20%	12.40%
65–74	16.70%	16.80%	16.60%
55–64	17.00%	17.10%	16.90%
45–54	17.10%	16.70%	17.40%
35–44	15.90%	17.00%	14.90%
25–34	11.70%	12.30%	11.20%
15–24	7.60%	7.00%	8.20%
Sex			
Man	49.80%	100%	-
Woman	50.20%	-	100%
Education level			
Obligatory schooling	13.70%	12.00%	15.30%
Secondary degree	45.00%	42.40%	47.60%
Tertiary degree	40.80%	45.10%	36.50%
(Missing)	0.50%	0.40%	0.60%
Home ownership			
Renter	52.80%	53.40%	52.30%
Owner	45.20%	44.70%	45.70%
Other	1.60%	1.50%	1.60%
(Missing)	0.40%	0.40%	0.40%
Civil status			
Single	36.70%	39.80%	33.60%
Married	48.40%	49.30%	47.50%
Divorced	10.50%	8.60%	12.40%
Widowed	4.40%	2.30%	6.60%
Nationality			
Swiss since birth	64.40%	63.80%	65.00%
Naturalised Swiss	12.50%	11.30%	13.70%
Foreigner	23.00%	24.80%	21.30%
(Missing)	0.00%	0.00%	0.00%
Urbanity			
Urban	61.80%	61.20%	62.40%
Intermediate	21.70%	22.10%	21.30%
Rural	16.50%	16.80%	16.30%
Linguistic region			
German	71.40%	72.40%	70.50%
French	24.20%	23.60%	24.80%
Italian	4.40%	4.00%	4.70%
Household size			
1	20.00%	18.80%	21.20%
>=2	80.00%	81.20%	78.80%
**Health-related characteristics**			
Self-rated health			
Good or very good	85.90%	86.80%	85.00%
Average	11.40%	10.50%	12.40%
Bad or very bad	2.60%	2.70%	2.60%
(Missing)	0.10%	0.10%	0.00%
Chronic condition or long-term health issue			
Yes	35.10%	32.70%	37.40%
No	64.60%	67.00%	62.20%
(Missing)	0.30%	0.30%	0.40%
Medication use (last 7 days)			
Yes	54.30%	50.40%	58.20%
No	45.70%	49.60%	41.80%
(Missing)	0.00%	0.00%	0.00%
Medical consultation (last 12 months)			
Yes	82.70%	75.80%	89.50%
No	17.30%	24.10%	10.50%
(Missing)	0.10%	0.10%	0.00%
Weekly physical activity			
Inactive	7.60%	6.70%	8.50%
Partially active	15.50%	13.80%	17.10%
Active	74.90%	77.60%	72.20%
(Missing)	2.00%	1.90%	2.10%
Smoking use			
Never smoker	53.70%	48.80%	58.60%
Former smoker	22.00%	23.90%	20.10%
Smoker	24.30%	27.30%	21.30%
(Missing)	0.00%	0.00%	0.00%

**Fig. 1:**
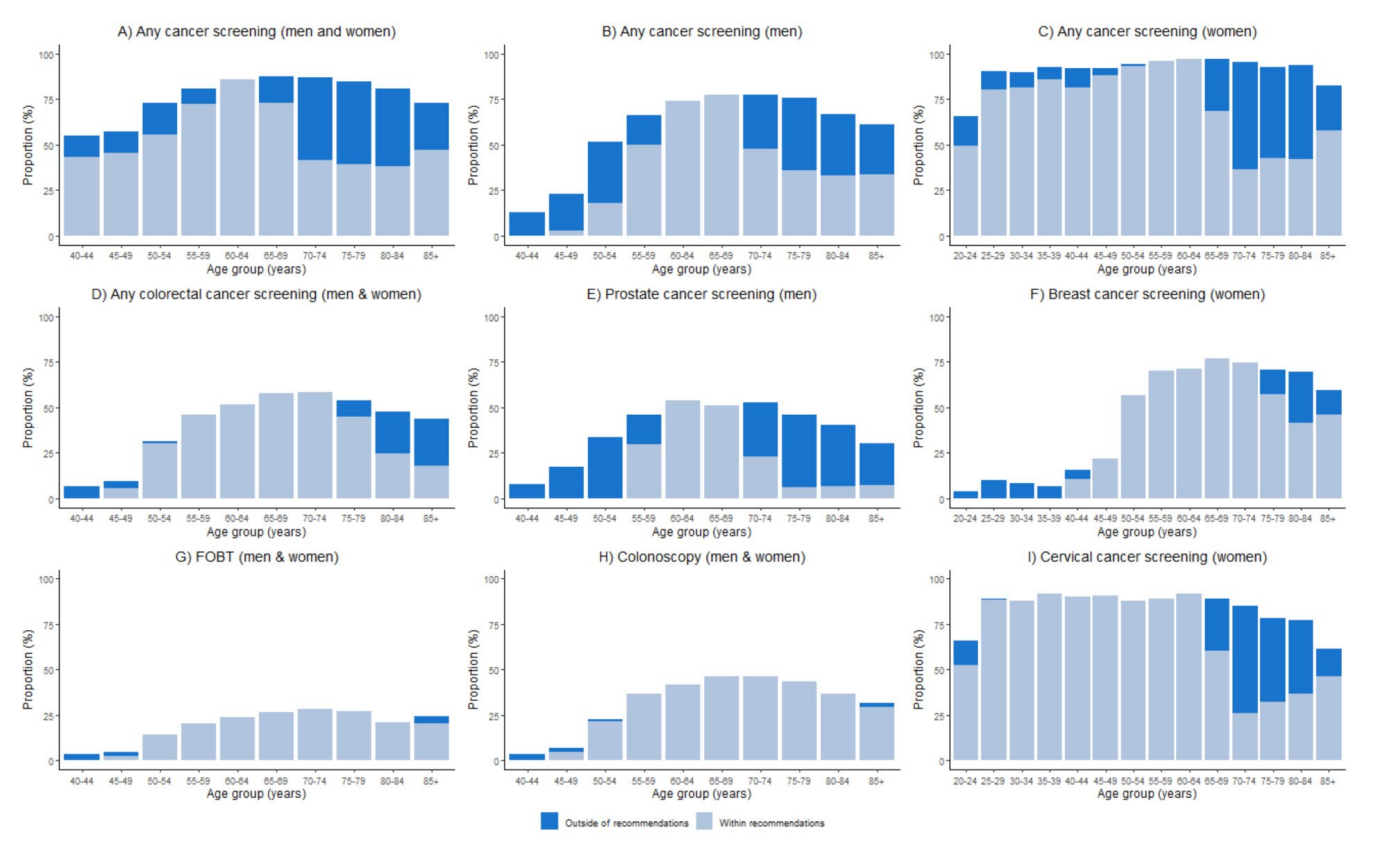
Visual overview of cancer screening use according to USPSTF **A**, **B**, and **C** recommendations (weighted proportions) Abbreviations: FOBT, fecal occult blood test; PSA, prostate-specific antigen test. For any cancer screening and any colorectal cancer screening, not screened corresponds to individuals who have never undergone a cancer screening test or all of their most recent tests were for non-preventive purposes, within recommendations corresponds to individuals who have undergone any cancer screening (or any colorectal cancer screening) only within recommendations, while outside of recommendations corresponds to individuals who have undergone cancer screening (or any colorectal cancer screening) which includes at least one last screening test which was outside of recommendations. For FOBT, colonoscopy, mammography, uterine smear, and PSA or rectal exam, not screened corresponds to individuals who have never undergone the corresponding cancer screening test or individuals whose most recent corresponding test was for non-preventive purposes, within recommendations corresponds to individuals who have undergone the corresponding cancer screening modality and their last test was within recommendations, while outside of recommendations corresponds to individuals who have undergone the corresponding cancer screening modality and their last test was outside of recommendations

Weighted proportions of cancer screening according to USPSTF recommendations among older adults 75+ years old, middle-aged adults 40–59 years old, and younger-aged women 20–39 years old are reported in Table 2. Weighted proportions across specific age-strata are reported in Table 3; Fig. 1 for USPSTF A, B, and C recommendations, and in Table S1 and Figure S3 for USPSTF A and B recommendations.

**Table 2:** Proportions of cancer screening use according to USPSTF recommendations among older-, middle-, and younger-aged adults (weighted proportions)

Screening engagement according to recommendations	Any cancer screening (men and women)			Colorectal cancer screening (men and women)			Breast cancer screening (women)	Cervical cancer screening (women)	Prostate cancer screening (men)
	All	Men	Women	Any	FOBT	Colonoscopy	Mammography	Uterine smear	PSA or rectal exam
** 75+ y/o**									
Not screened Within A, B, and C recommendations Outside of A, B, and C recommendations	18.9% 40.8% 40.2%	30.5% 34.4% 35.1%	9.3% 46.2% 44.5%	50.3% 48.4% 1.3%	75.3% 23.8% 0.9%	61.2% 38.3% 0.5%	32.3% 49.9% 17.8%	25.9% 37.1% 37.1%	59.5% 6.5% 34.0%
Not screened Within A and B recommendations Outside of A and B recommendations	18.9% 30.7% 50.4%	30.5% 19.0% 50.5%	9.3% 40.5% 50.2%	50.3% 32.4% 17.3%	75.3% 15.4% 9.2%	61.2% 27.8% 11.0%	32.3% 49.9% 17.8%	25.9% 37.1% 37.1%	59.5% 0.0% 40.5%
**40-59y/o**									
Not screened Within A, B, and C recommendations Outside of A, B, and C recommendations	33.3% 54.4% 12.3%	60.9% 18.3% 20.8%	6.2% 89.8% 4.0%	76.5% 20.6% 2.9%	89.3% 9.3% 1.4%	82.3% 15.9% 1.7%	59.3% 39.4% 1.3%	10.5% 89.5% 0.0%	73.4% 0.0% 26.6%
Not screened Within A and B recommendations Outside of A and B recommendations	33.3% 45.9% 20.9%	60.9% 10.7% 28.5%	6.2% 80.4% 13.4%	76.5% 20.6% 2.9%	89.3% 9.3% 1.4%	82.3% 15.9% 1.7%	59.3% 29.5% 11.2%	10.5% 89.5% 0.0%	73.4% 0.0% 26.6%
**20-39y/o**									
Not screened Within A, B, and C recommendations Outside of A, B, and C recommendations	- - -	- - -	13.9% 76.2% 9.9%	- - -	- - -	- - -	92.6% 0.0% 7.4%	15.1% 82.2% 2.7%	- - -
Not screened Within A and B recommendations Outside of A and B recommendations	- - -	- - -	13.9% 76.2% 9.9%	- - -	- - -	- - -	92.6% 0.0% 7.4%	15.1% 82.2% 2.7%	- - -

Abbreviations: y/o, years old; FOBT, fecal occult blood test; PSA, prostate-specific antigen test. For any cancer screening and any colorectal cancer screening, not screened corresponds to individuals who have never undergone a cancer screening test or all of their most recent tests were for non-preventive purposes, within recommendations corresponds to individuals who have undergone any cancer screening (or any colorectal cancer screening) only within recommendations, while outside of recommendations corresponds to individuals who have undergone cancer screening (or any colorectal cancer screening) which includes at least one last screening test which was outside of recommendations. For FOBT, colonoscopy, mammography, uterine smear, and PSA or rectal exam, not screened corresponds to individuals who have never undergone the corresponding cancer screening test or individuals whose most recent corresponding test was for non-preventive purposes, within recommendations corresponds to individuals who have undergone the corresponding cancer screening modality and their last test was within recommendations, while outside of recommendations corresponds to individuals who have undergone the corresponding cancer screening modality and their last test was outside of recommendations

**Table 3:** Proportions of cancer screening use according to USPSTF A, B, and C recommendations (weighted proportions)

	Any cancer screening (men and women)			Colorectal cancer screening (recommended to adults 45-85y/o)			Breast cancer screening (recommended to women 40-74y/o)	Cervical cancer screening (recommended to women 21-65y/o)	Prostate cancer screening (recommended to men 55-69y/o)
Age group (years) and screening engagement according to recommendations	All	Men	Women	Any	FOBT	Colonoscopy	Mammography	Uterine smear	PSA or rectal exam
85+									
Not screened	26.90%	38.70%	17.40%	56.50%	75.70%	68.00%	40.70%	38.50%	69.70%
Within recommendations	47.10%	33.60%	58.00%	38.10%	20.30%	29.70%	45.80%	46.40%	7.40%
Outside of recommendations	26.00%	27.80%	24.60%	5.40%	4.00%	2.20%	13.60%	15.10%	22.90%
80–84									
Not screened	19.20%	33.40%	6.30%	52.30%	78.80%	63.00%	30.30%	22.90%	59.90%
Within recommendations	38.00%	33.10%	42.40%	47.70%	21.20%	37.00%	41.30%	36.90%	6.60%
Outside of recommendations	42.80%	33.50%	51.20%	0.00%	0.00%	0.00%	28.40%	40.10%	33.50%
75–79									
Not screened	14.90%	24.50%	7.10%	46.00%	72.90%	56.60%	29.40%	21.40%	54.20%
Within recommendations	39.50%	35.70%	42.70%	54.00%	27.10%	43.40%	57.20%	32.50%	5.90%
Outside of recommendations	45.60%	39.90%	50.20%	0.00%	0.00%	0.00%	13.40%	46.10%	39.90%
70–74									
Not screened	12.90%	22.50%	4.50%	41.80%	71.80%	53.60%	25.20%	14.70%	47.20%
Within recommendations	41.80%	47.80%	36.50%	58.20%	28.20%	46.40%	74.80%	26.30%	23.00%
Outside of recommendations	45.30%	29.80%	59.00%	0.00%	0.00%	0.00%	0.00%	59.00%	29.80%
65–69									
Not screened	12.10%	22.20%	3.00%	42.10%	73.40%	53.90%	23.40%	11.00%	49.00%
Within recommendations	73.00%	77.80%	68.60%	57.90%	26.60%	46.10%	76.60%	60.60%	51.00%
Outside of recommendations	14.90%	0.00%	28.40%	0.00%	0.00%	0.00%	0.00%	28.40%	0.00%
60–64									
Not screened	14.10%	25.70%	2.80%	48.50%	76.00%	58.40%	28.90%	7.90%	46.30%
!Within recommendations	85.90%	74.30%	97.20%	51.50%	24.00%	41.60%	71.10%	92.10%	53.70%
Outside of recommendations	0.00%	0.00%	0.00%	0.00%	0.00%	0.00%	0.00%	0.00%	0.00%
55–59									
Not screened	19.00%	33.90%	3.70%	53.90%	79.80%	63.40%	30.00%	10.80%	54.10%
Within recommendations	72.70%	49.90%	96.30%	46.10%	20.20%	36.60%	70.00%	89.20%	29.70%
Outside of recommendations	8.20%	16.20%	0.00%	0.00%	0.00%	0.00%	0.00%	0.00%	16.20%
50–54									
Not screened	27.00%	48.20%	5.70%	68.50%	85.70%	77.10%	43.60%	11.90%	66.60%
Within recommendations	55.60%	18.20%	93.40%	30.30%	14.30%	21.80%	56.40%	88.10%	0.00%
Outside of recommendations	17.40%	33.60%	0.90%	1.10%	0.00%	1.10%	0.00%	0.00%	33.40%
45–49									
Not screened	42.60%	77.10%	7.90%	90.70%	95.40%	92.80%	78.10%	9.30%	82.50%
Within recommendations	45.40%	2.90%	88.20%	5.20%	2.40%	4.80%	21.90%	90.70%	0.00%
Outside of recommendations	12.00%	20.00%	4.00%	4.10%	2.10%	2.40%	0.00%	0.00%	17.50%
40–44									
Not screened	44.90%	87.30%	7.60%	93.60%	96.40%	96.60%	84.70%	10.10%	92.50%
Within recommendations	43.50%	0.00%	81.70%	0.00%	0.00%	0.00%	10.50%	89.90%	0.00%
Outside of recommendations	11.60%	12.70%	10.70%	6.40%	3.60%	3.40%	4.80%	0.00%	7.50%
35–39									
Not screened	-	-	7.30%	-	-	-	93.20%	8.30%	-
Within recommendations	-	-	85.90%	-	-	-	0.00%	91.70%	-
Outside of recommendations	-	-	6.80%	-	-	-	6.80%	0.00%	-
30–34									
Not screened	-	-	10.10%	-	-	-	91.80%	11.90%	-
Within recommendations	-	-	81.70%	-	-	-	0.00%	88.10%	-
Outside of recommendations	-	-	8.20%	-	-	-	8.20%	0.00%	-
25–29									
Not screened	-	-	9.30%	-	-	-	89.90%	11.10%	-
Within recommendations	-	-	80.40%	-	-	-	0.00%	88.70%	-
Outside of recommendations	-	-	10.30%	-	-	-	10.10%	0.20%	-
20–24									
Not screened	-	-	34.00%	-	-	-	96.20%	34.00%	-
Within recommendations	-	-	49.70%	-	-	-	0.00%	52.70%	-
Outside of recommendations	-	-	16.30%	-	-	-	3.80%	13.30%	-

Abbreviations: FOBT, fecal occult blood test; PSA, prostate-specific antigen test. For any cancer screening and any colorectal cancer screening, not screened corresponds to individuals who have never undergone a cancer screening test or all of their most recent tests were for non-preventive purposes, within recommendations corresponds to individuals who have undergone any cancer screening (or any colorectal cancer screening) only within recommendations, while outside of recommendations corresponds to individuals who have undergone cancer screening (or any colorectal cancer screening) which includes at least one last screening test which was outside of recommendations. For FOBT, colonoscopy, mammography, uterine smear, and PSA or rectal exam, not screened corresponds to individuals who have never undergone the corresponding cancer screening test or individuals whose most recent corresponding test was for non-preventive purposes, within recommendations corresponds to individuals who have undergone the corresponding cancer screening modality and their last test was within recommendations, while outside of recommendations corresponds to individuals who have undergone the corresponding cancer screening modality and their last test was outside of recommendations

### Any cancer screening

In total, 40.2% of older adults 75+ years old have undergone cancer screening outside of USPSTF A, B, and C recommendations, and 50.4% outside of A and B recommendations. Screening outside of A, B, and C recommendations was more frequent among older women than older men (44.5% vs. 35.1%), while screening outside of A and B recommendations was similar between sexes (50.2% vs. 50.5%). Among specific age-strata of adults aged 65+, proportions of screening outside of A, B, and C recommendations ranged from 14.9% at ages 65–69 to 45.6% at ages 75–79 (39.1-56.1% outside of A and B recommendations). Among middle-aged adults 40–59, 12.3% (men: 20.8%; women: 4.0%) had been screened outside A, B, and C recommendations, and 20.9% outside of A and B recommendations (men: 28.5%; women: 13.4%). The proportion of women aged 20–39 who underwent screening outside of recommendations was 9.9%.

### Colorectal cancer screening

Proportions of any colorectal cancer screening outside of recommendations among adults aged 75+ was 1.3% for A, B, and C recommendations and 17.3% for A and B recommendations. Colonoscopies outside of A and B recommendations were more frequent than FOBTs outside of recommendations (adults 75+: 11.0% vs. 9.2%).

### Breast cancer screening

For mammography, 17.8% of women 75+ reported having been screened outside of USPSTF recommendations for both A, B, and C and A and B recommendations. Women aged 80–84 undertook mammograms outside of recommendations at the highest frequency of any age-strata of older women at 28.4% (75–79: 13.4%; 85+: 13.6%). Among middle-aged women 40–59, screening outside of recommendations was 1.3% for A, B, and C recommendations and 11.2% for A and B recommendations. Among younger-aged women 20–39, screening outside of recommendations was 7.4%. In more specific age-strata of middle- and younger-aged women, screening outside of recommendations was highest in the 25–29 age group for A, B, and C recommendations (10.1%) and in the 45–49 age group for A and B recommendations (21.9%).

### Cervical cancer screening

Uterine smears outside of A, B, and C and A and B recommendations were frequently reported by older women aged 75+ (37.1%). Among more specific age-strata, proportions of screening outside of recommendations reached 59.0% (women aged 70–74). Among women 20–24 years old, screening outside of recommendations was 13.3%.

### Prostate cancer screening

The proportion of older men 75+ who have undergone prostate cancer screening outside of recommendations was 34.0% for A, B, and C recommendations and 40.5% for A and B recommendations. Within specific age-strata, the highest rate of screening outside of recommendations was observed among men aged 75–79 for A, B, and C recommendations (39.9%), and among men aged 60–64 for A and B recommendations (53.7%). Screening outside of recommendations was also common among middle-aged men 40–59 (26.6%), with a high of 33.4% in the 50–54 age-strata for A, B, and C recommendations.

### Estimated misclassification of age at screening

Tables S2 and S3 provide estimates of the proportion of participants misclassified as having been screened within recommendations for specific age-strata and cancer screening modalities. This misclassification was generally not present or modest for colorectal cancer and prostate cancer screening (0.0-6.5% in different age-strata). For breast cancer and cervical cancer screening, this misclassification was substantial. Among women 75+, the estimated proportion of misclassified participants was 6.6% for mammography and 18.7% for uterine smears. Among women 85+, these proportions were 22.2% and 27.0%, respectively. If we account for this misclassification (see detailed analyses by age and types of screening in Tables S4, S5, and S6 and Figures S4 and S5), the proportion of women 75+ who have been screened outside of USPSTF recommendations (both A, B, and C and A and B) was 25.5% for breast cancer and 58.6% for cervical cancer screening, while for prostate cancer the proportion of men 75+ who have been screened outside of USPSTF A, B, and C recommendations was 39.7%.

## Discussion

This study sought to describe the frequency of cancer screening outside of USPSTF recommended age guidelines in Switzerland. Our results show that cancer screening outside of recommendations was very common. Among older adults 75+ years of age, 40.2% engaged in a screening test outside of USPSTF A, B, and C recommendations, and 50.4% outside of A and B recommendations. Cervical cancer screening was generally the most frequently used outside of A, B, and C recommendations across age groups, followed by prostate, breast, and colorectal cancer screening. Middle- and younger-aged adults are also shown to frequently undergo cancer screening outside of recommendations, with 12.3% of adults aged 40–59 and 7.4% of younger women aged 20–39 having undergone screening outside of A, B, and C recommendations. Low-value cancer screening is therefore frequent in the population of Switzerland.

These results coincide with the findings of studies from the United States, Canada, and France [11–19], where high frequencies of cancer screening outside of recommendations has also been observed. Most notably, one study found that the proportion of community-dwelling older adults who have undergone colorectal, cervical, and breast cancer screening outside of recommendations ranged between 45.8 and 74.1% [11]. Further, cancer screening outside of recommendations, namely in the form of screening more frequently than recommended, has been observed for cervical cancer screening in Europe– including Switzerland specifically [27–29]. Existing literature also provides potential explanations for why cancer screening outside of recommendations is so frequent. For instance, research among older adults has found that the reasons why cancer screening recommendations have age cut-offs are not well known or understood [30], and that many believe that undergoing screening outside of recommended ages is okay [31]. Correspondingly, studies have also found that people – including clinicians – generally overestimate the benefits of screening while underestimating its harms [1, 30, 32, 33], with one qualitative study finding that some older adults have never been made aware of any potential risks associated with screening [30]. It is therefore likely that many patients and clinicians are making decisions about cancer screening that are not appropriately informed.

Our results are concerning as it represents low-value care, which refers to care that brings minimal-to-no benefit to patients [3] and where harms are not outweighed by benefits at a reasonable cost according to existing evidence [6]. Cancer screening outside of evidence-based recommendations thereby falls within this category of low-value care, as the lack of recommendations for screening in certain age groups stems from high-quality evidence not supporting its use. In turn, the very high frequency of screening outside of recommendations indicates that many cancer screening practices in Switzerland are not based in evidence and, therefore, that a significant proportion of the population is being unnecessarily exposed to potential harms of screening. These harms include false positives, false negatives, unnecessary emotional stress, complications, and overdiagnosis – where inconsequential cancers that would have never become clinically significant in a person’s lifetime had they not undergone screening are diagnosed – which can then lead to overtreatment [1, 34–36].With that said, while population-based cancer screening guidelines – such as those from the USPSTF – represent what people should be doing based on averaged benefits and harms, evaluating individual benefit of cancer screening entails considerable complexity [37]. In turn, simply because someone falls outside of the recommended age guidelines does not necessarily mean they will not benefit from being screened [38]. Many have therefore called for a personalised approach to cancer screening, such as risk-based screening or screening according to lag-time to benefit and life expectancy, with the assumption that some subgroups of people will benefit while others will not [38–40]. These approaches provide alternatives to population-based recommendations that may be promising for older adults given the absence of high-quality evidence on screening efficacy among those 75 years of age and above, which stems from their common exclusion from cancer screening trials [38]. However, these personalised approaches to cancer screening must be tested in trials and raise major methodological and practical issues, including the ability to reliably assess risk and estimate benefit at a subgroup level [37]. This highlights the important role of shared decision making to ensure that evidence-based screening practices also take into account patient values and preferences [41].

### Limitations

This study is subject to several limitations. First, results may not be nationally representative as the 2022 Swiss Health Survey had a participation rate of 36.2%. While weights improving national representativeness were applied to reported proportions, screening frequency within our sample is likely higher than within our target population due to healthy volunteer bias [42]. Second, while the USPSTF recommendations can reasonably be applied to Switzerland, they are ultimately not developed specifically for the Swiss context. Third, cancer screening use data are self-reported by participants, which is prone to inaccuracy and likely overestimates screening use [43]. Third, inaccuracies in self-reported time and reason of testing may have led to misclassification of screening use according to recommendations. Fourth, we were unable to determine whether participants who utilised screening tests for non-preventive/screening purposes had ever undergone those same tests for preventive/screening purposes. These participants were, therefore, classified as having not been screened for the corresponding screening modalities. While this results in underestimated proportions of screening outside of recommendations, their exclusion from the study would have resulted in an overestimate of these same proportions.

Additional limitations include that the timing of cancer screening variables within the Swiss Health Survey provides a time window of when screening tests were last undertaken rather than a specific date. Accordingly, as explained in the methods, to ensure that we did not misclassify participants as having been screened outside of recommendations, our analysis underestimates the frequency of screening outside of recommendations. We therefore sought to estimate the size of this misclassification by analysing data from the 2017 Swiss Health Survey in which the ascertainment of time of last screening variables allowed for age at screening to be calculated more accurately. The results of these calculations suggest that the true frequency of screening outside of recommendations is likely higher than estimated within the main analysis of this study.

### Implications

Our findings indicate a need for increasing awareness among clinicians about the importance of evidence-based cancer screening recommendations and the harms associated with administering screening tests outside of such recommendations. Concurringly, policymakers should introduce policies to deter the use of screening tests not supported by evidence and introduce educational campaigns informing the population about the potential harms of screening and that the benefits of non-recommended screenings are minimal or non-existent [3]. The Choosing Wisely campaign – established in Switzerland under the name Smarter Medicine [44] – provides one example of a platform that could drive reductions in screening use outside of recommendations [45, 46]. Future research on this topic is also needed and should focus on describing the extent of cancer screening outside of recommendations in other high-income countries, as our results in Switzerland and similar results in the United States, Canada, and France [11–19] provide reason to believe that such screening is likely frequent. Following a healthcare monitoring and improvement approach [47], studies should also identify key populations and healthcare practitioners among whom screening outside of recommendations is most frequent, and the factors that drive such non-recommended screening use. This can help inform the development of interventions to de-implement and deter low-value cancer screening, which urgently need to be developed.

## Conclusion

Low-value cancer screening is very common, as evidenced by the high frequency of cancer screening outside of age recommendations. This problem is predominantly common among older adults, with 40.2% of adults 75+ years of age having been screened outside of USPSTF A, B, and C recommendations. These results indicate that cancer screening practices are often not evidence-based, with many individuals being unnecessarily exposed to the potential harms of screening despite evidence not showing that they can experience a meaningful benefit. Interventions aimed at deterring the use of this form of low-value care therefore need to be developed and implemented.

## Electronic supplementary material

Below is the link to the electronic supplementary material.

Supplementary Material 1

## Data Availability

Data from the Swiss Health Survey 2022 are available for a fee (400 Swiss Francs, plus 7.7% tax) and users must request permission from the Swiss Federal Statistical Office (sgb@bfs.admin.ch). Data must be destroyed after 5 years.
