# Supplementary material for: Cancer screening outside of age recommendations: a population-based study
Source: BMC Public Health. 2025 May 6;25:1660. doi: 10.1186/s12889-025-22848-4 (PMC12054051; doi:10.1186/s12889-025-22848-4)
Supplement: Supplementary file 1 — Supplementary Material 1 [file 12889_2025_22848_MOESM1_ESM.docx]

**Supplementary Material**

**Cancer screening outside of age recommendations: a population-based study**

**Authors:** Frerik Smit, Vladimir Jolidon, Bernadette WA van der Linden, Nicolas Rodondi, Stéphane Cullati, Arnaud Chiolero

Table of Contents

[Figure S1 – Flowchart of participant inclusion 2](#_Toc184112459)

[Ascertainment methods of outcome variables 3](#_Toc184112460)

[Ascertainment methods of participant characteristics variables 4](#_Toc184112461)

[Figure S2 – Summary map of USPSTF cancer screening guidelines (prior to 2022) 5](#_Toc184112462)

[Statistical analysis for estimating age at screening misclassification 6](#_Toc184112463)

[Table S1 – Proportions of cancer screening use according to USPSTF A and B recommendations (weighted proportions) 8](#_Toc184112464)

[Figure S3 – Visual overview of cancer screening use according to USPSTF A and B recommendations (weighted proportions) 10](#_Toc184112465)

[Table S2 – Estimated proportion of participants misclassified as having been screened within USPSTF A, B, and C recommendations (unweighted proportions) 12](#_Toc184112466)

[Table S3 – Estimated proportion of participants misclassified as having been screened within USPSTF A and B recommendations (unweighted proportions) 13](#_Toc184112467)

[Table S4 – Proportions of cancer screening use according to USPSTF recommendations among older adults 75+ years of age (unweighted proportions corrected for estimated age at screening misclassification) 14](#_Toc184112468)

[Table S5 – Proportions of cancer screening use according to USPSTF A, B, and C recommendations (unweighted proportions corrected for estimated age at screening misclassification) 15](#_Toc184112469)

[Table S6 – Proportions of cancer screening use according to USPSTF A and B recommendations (unweighted proportions corrected for estimated misclassification error) 17](#_Toc184112470)

[Figure S4 – Visual overview of cancer screening use according to USPSTF A, B, and C recommendations with estimate of misclassification error (unweighted proportions) 19](#_Toc184112471)

[Figure S5 – Visual overview of cancer screening use according to USPSTF A and B recommendations with estimate of misclassification error (unweighted proportions) 21](#_Toc184112472)

[Table S7 – Strengthening the reporting of observational studies in epidemiology (STROBE) checklist 23](#_Toc184112473)

# Figure S1 – Flowchart of participant inclusion


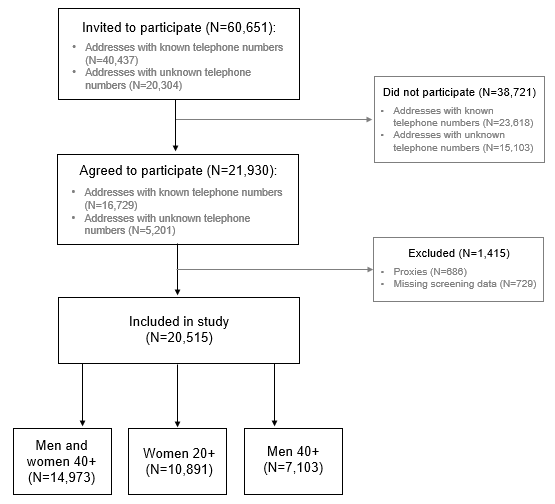


# Ascertainment methods of outcome variables

For breast cancer screening, women 20 years of age and above (20+) were asked “Have you undergone a mammogram?”, with participants responding in the affirmative subsequently being asked “When was your last mammogram?” and “What was the reason for your last mammogram?”. For colorectal cancer screening, all adults 40 years of age and above (40+) were asked both “Have you undergone a test to detect blood in faeces?” and “Have you undergone a colonoscopy?”. Participants who answered yes to either question were subsequently asked when they last underwent a faecal occult blood test (FOBT) and/or colonoscopy, as well as the reason for said test(s). For cervical cancer screening, women 20+ years of age were asked “Have you undergone a uterine smear?” and, if yes, “When was your last uterine smear?”. For prostate cancer screening, men 40+ years of age were asked “Have you undergone a prostate exam?” and, if yes, “How was your last prostate exam undertaken?” (rectal exam, PSA-test, both), “What was the reason of your last prostate exam?”, and “When was your last prostate exam?”. For questions related to the reason of undergoing a specified test, participants were given the option of answering either “Preventive exam/check-up”, “Diagnostic test following symptoms”, “Follow-up test following results of prior test”, or “Other”. For questions related to the time of their last mammogram, FOBT, uterine smear, or prostate exam, participants could either answer “Within the last 12 months”, “1 year to less than 2 years ago, “2 years to less than 3 years ago”, “3 years to less than 5 years ago”, or “More than 5 years ago”. Meanwhile, for the time of last colonoscopy question, participants could either answer “Within the last 12 months”, “1 year to less than 5 years ago”, “5 years to less than 10 years ago”, or “More than 10 years ago”.

# Ascertainment methods of participant characteristics variables

Age was ascertained through self-reported date of birth. Sex was ascertained by asking participants “Who are you?” (Man/Woman). For education level, participants were asked “What is the highest level of training that you have achieved?”, with responses subsequently coded into either “Obligatory schooling”, “Secondary degree”, or “Tertiary degree” according to the International Standard Classification of Education. Home ownership was ascertained by asking participants whether they or someone else in their household are a renter or owner of the household they are living in. Participants’ civil status and nationality were obtained from cantonal registries. Urbanity and linguistic region were constructed according to a geographic overview of Switzerland from the Office of Federal Statistics using participants’ commune of residence. Household size was constructed as a continuous variable through a number of items that asked participants specific questions about persons living in the same household at least 4 days per week, which we subsequently collapsed into a binary categorical variable of 1 and greater than or equal to 2. Self-rated health was ascertained through the question “How is your general health state? Is it...” either “Very good”, “Good”, “Average”, “Bad”, or “Very bad”, which we collapsed into three categories: “Good or very good”, “Average”, and “Bad or very bad”. The physical activity variable was constructed by asking participants a number of questions related to the type, frequency, and duration of the physical activities they engaged in within the past week, which was categorised into three categories: “Inactive” (less than 30 minutes of exercise or exercising less than once), “Partially active” (30-149 minutes of exercise or exercising once), and “Active” (greater than 149 minutes of exercise or exercising at least twice). For smoking use, participants were asked if they actively consume tobacco and, if not, whether they have ever smoked for more than 6 months. Subsequently, they were categorised into “Never smoker”, “Former smoker”, and “Smoker”. Finally, for the remaining health-related characteristics, participants were asked “Do you have a chronic or long-term health issue?”, “Have you taken any medications over the course of the last 7 days?”, and “Have you been to a doctor, including your family doctor, over the course of the last 12 months, not including the dentist?”.

# Figure S2 – Summary map of USPSTF cancer screening guidelines (prior to 2022)


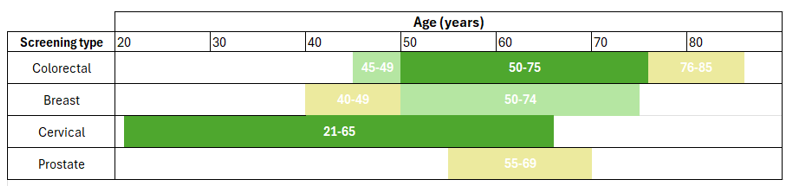


USPSTF recommendation grades:

| **A** | **B** | **C** |
| --- | --- | --- |

A: high certainty of substantial net benefit; B: high or moderate certainty of moderate net benefit; C: at least moderate certainty of small net benefit

# Statistical analysis for estimating age at screening misclassification

We estimated the size of age at screening misclassification using data from the 2017 wave of the SHS in several steps. First, for individual cancer screening modalities, we excluded participants with missing data on the year of their last screening test. Second, among participants who underwent individual screening tests explicitly for preventive purposes, we identified all participants 60+ years of age whose last test for said screening modality was 5+ years ago (10+ for colonoscopy). Third, among these participants, we calculated how recently participants’ tests were undertaken by subtracting the year of a screening test by the year the survey was conducted (2017). Fourth, we then calculated age at screening by subtracting this constructed recency of screening test variable from participants’ age. Fifth, participants were subsequently classified as having been screened within or outside of USPSTF recommendations in the same manner that was used for our main analysis. Sixth, we then calculated the proportion of participants 60+ years of age who screened 5+ years ago (10+ for colonoscopy) that had screened outside of recommendations within specific age strata. These proportions calculated from the 2017 SHS thereby provide an estimate of the proportion of participants 60+ years of age who screened 5+ years ago (10+ for colonoscopy) in the 2022 SHS that were misclassified as having been screened within recommendations instead of outside of recommendations.

We then extended this estimated age at screening misclassification to get an estimate of the total proportion of participants within specific age strata in the 2022 SHS that were misclassified as having been screened within recommendations for individual screening modalities. To do this, we first identified the total number of participants within specific age strata who screened 5+ years ago for specific screening modalities (10+ for colonoscopy). We then applied the estimated proportion of participants misclassified calculated afore to obtain an estimated specific total number and proportion of participants that were misclassified (Tables S2 and S3). The corrected proportion of participants who screened within recommendations was then calculated by subtracting the number of participants misclassified from the original number of participants classified as having been screened within recommendations. Correspondingly, the proportion of participants who were screened outside of recommendations was calculated by adding the number of participants misclassified from the original number of participants classified as having been screened outside of recommendations. These corrected proportions are reported in Tables S4, S5, and S6 and visually presented in Figures S3 and S4. Of note, these proportions are unweighted given that the calculation of weighted proportions was not possible as that would have necessitated the retroactive allocation of misclassification assignments to specific participants in the 2022 SHS.

# Table S1 – Proportions of cancer screening use according to USPSTF A and B recommendations (weighted proportions)

|  | **Any cancer screening (men and women)** | | | **Colorectal cancer screening (recommended to adults 45-75y/o)** | | | **Breast cancer screening (recommended to women 50-74y/o)** | **Cervical cancer screening (recommended to women 21-65y/o)** | **Prostate cancer screening (none recommended)** |
| --- | --- | --- | --- | --- | --- | --- | --- | --- | --- |
| **Age group (years) and screening engagement according to recommendations** | **All** | **Men** | **Women** | **Any** | **FOBT** | **Colonoscopy** | **Mammography** | **Uterine smear** | **PSA or rectal exam** |
| 85+  Not screened  Within recommendations  Outside of recommendations | 26.9%  32.5%  40.6% | 38.7%  14.2%  47.1% | 17.4%  47.1%  35.4% | 56.5%  18.0%  25.5% | 75.7%  13.6%  10.7% | 68.0%  11.9%  20.1% | 40.7%  45.8%  13.6% | 38.5%  46.4%  15.1% | 69.7%  0.0%  30.3% |
| 80-84  Not screened  Within recommendations  Outside of recommendations | 19.2%  25.6%  55.2% | 33.4%  15.3%  51.3% | 6.3%  34.8%  58.8% | 52.3%  24.5%  23.3% | 78.8%  9.1%  12.2% | 63.0%  22.4%  14.6% | 30.3%  41.3%  28.4% | 22.9%  36.9%  40.1% | 59.9%  0.0%  40.1% |
| 75-79  Not screened  Within recommendations  Outside of recommendations | 14.9%  33.1%  52.0% | 24.5%  23.8%  51.7% | 7.1%  40.6%  52.3% | 46.0%  44.6%  9.4% | 72.9%  20.4%  6.7% | 56.6%  39.1%  4.3% | 29.4%  57.2%  13.4% | 21.4%  32.5%  46.1% | 54.2%  0.0%  45.8% |
| 70-74  Not screened  Within recommendations  Outside of recommendations | 12.9%  31.0%  56.1% | 22.5%  24.8%  52.8% | 4.5%  36.5%  59.0% | 41.8%  58.2%  0.0% | 71.8%  28.2%  0.0% | 53.6%  46.6%  0.0% | 25.2%  74.8%  0.0% | 14.7%  26.3%  59.0% | 47.2%  0.0%  52.8% |
| 65-69  Not screened  Within recommendations  Outside of recommendations | 12.1%  48.7%  39.1% | 22.2%  26.8%  51.0% | 3.0%  68.6%  28.4% | 42.1%  57.9%  0.0% | 73.4%  26.6%  0.0% | 53.9%  46.1%  0.0% | 23.4%  76.6%  0.0% | 11.0%  60.6%  28.4% | 49.0%  0.0%  51.0% |
| 60-64  Not screened  Within recommendations  Outside of recommendations | 14.1%  59.3%  26.6% | 25.7%  20.5%  53.7% | 2.8%  97.2%  0.0% | 48.5%  51.5%  0.0% | 76.0%  24.0%  0.0% | 58.4%  46.1%  0.0% | 28.9%  71.1%  0.0% | 7.9%  92.1%  0.0% | 46.3%  0.0%  53.7% |
| 55-59  Not screened  Within recommendations  Outside of recommendations | 19.0%  57.7%  23.3% | 33.9%  20.2%  45.9% | 3.7%  96.3%  0.0% | 53.9%  46.1%  0.0% | 79.8%  20.2%  0.0% | 63.4%  36.6%  0.0% | 30.0%  70.0%  0.0% | 10.8%  89.2%  0.0% | 54.1%  0.0%  45.9% |
| 50-54  Not screened  Within recommendations  Outside of recommendations | 27.0%  51.8%  21.2% | 48.2%  18.2%  33.6% | 5.7%  85.7%  8.6% | 68.5%  30.3%  1.1% | 85.7%  14.3%  0.0% | 77.1%  21.8%  1.1% | 43.6%  48.8%  7.6% | 11.9%  88.1%  0.0% | 66.6%  0.0%  33.4% |
| 45-49  Not screened  Within recommendations  Outside of recommendations | 42.6%  35.0%  22.4% | 77.1%  2.9%  20.0% | 7.8%  67.3%  24.9% | 90.7%  5.2%  4.1% | 95.4%  2.4%  2.1% | 92.8%  4.8%  2.4% | 78.1%  0.0%  21.9% | 9.3%  90.7%  0.0% | 82.5%  0.0%  17.5% |
| 40-44  Not screened  Within recommendations  Outside of recommendations | 44.9%  38.4%  16.6% | 87.3%  0.0%  12.7% | 7.6%  72.2%  20.2% | 93.6%  0.0%  6.4% | 96.4%  0.0%  3.6% | 96.6%  0.0%  3.4% | 84.7%  0.0%  15.3% | 10.1%  89.9%  0.0% | 92.5%  0.0%  7.5% |
| 35-39  Not screened  Within recommendations  Outside of recommendations | -  -  - | -  -  - | 7.3%  85.9%  6.8% | -  -  - | -  -  - | -  -  - | 93.2%  0.0%  6.8% | 8.3%  91.7%  0.0% | -  -  - |
| 30-34  Not screened  Within recommendations  Outside of recommendations | -  -  - | -  -  - | 10.1%  81.7%  8.2% | -  -  - | -  -  - | -  -  - | 91.8%  0.0%  8.2% | 11.9%  88.1%  0.0% | -  -  - |
| 25-29  Not screened  Within recommendations  Outside of recommendations | -  -  - | -  -  - | 9.3%  80.4%  10.3% | -  -  - | -  -  - | -  -  - | 89.9%  0.0%  10.1% | 11.1%  88.7%  0.2% | -  -  - |
| 20-24  Not screened  Within recommendations  Outside of recommendations | -  -  - | -  -  - | 34.0%  49.7%  16.3% | -  -  - | -  -  - | -  -  - | 96.2%  0.0%  3.8% | 34.0%  52.7%  13.3% | -  -  - |

Abbreviations: y/o, years old; FOBT, fecal occult blood test; PSA, prostate-specific antigen test. For any cancer screening and any colorectal cancer screening, not screened corresponds to individuals who have never undergone a cancer screening test or all of their most recent tests were for non-preventive purposes, within recommendations corresponds to individuals who have undergone any cancer screening (or any colorectal cancer screening) only within recommendations, while outside of recommendations corresponds to individuals who have undergone cancer screening (or any colorectal cancer screening) which includes at least one last screening test which was outside of recommendations. For FOBT, colonoscopy, mammography, uterine smear, and PSA or rectal exam, not screened corresponds to individuals who have never undergone the corresponding cancer screening test or individuals whose most recent corresponding test was for non-preventive purposes, within recommendations corresponds to individuals who have undergone the corresponding cancer screening modality and their last test was within recommendations, while outside of recommendations corresponds to individuals who have undergone the corresponding cancer screening modality and their last test was outside of recommendations.

# Figure S3 – Visual overview of cancer screening use according to USPSTF A and B recommendations (weighted proportions)


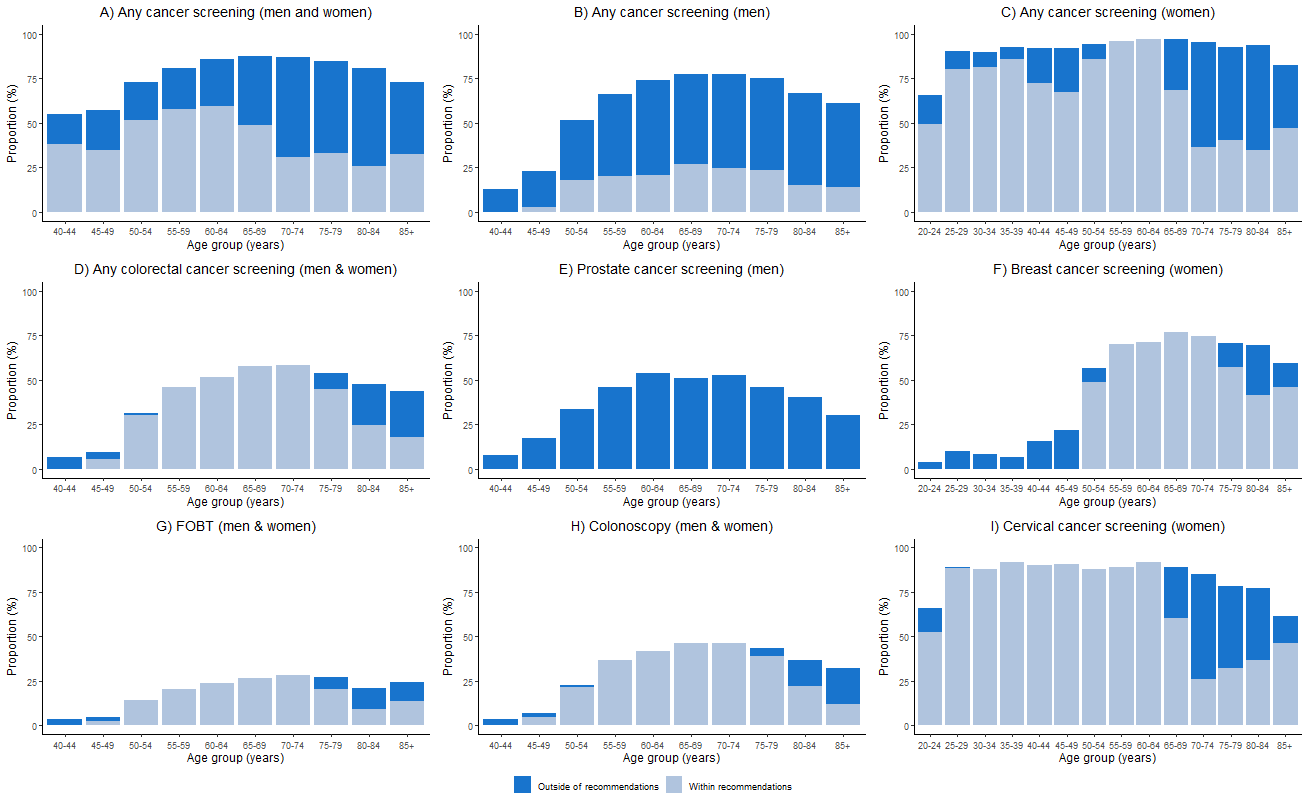


Abbreviations: FOBT, fecal occult blood test; PSA, prostate-specific antigen test. For any cancer screening and any colorectal cancer screening, not screened corresponds to individuals who have never undergone a cancer screening test or all of their most recent tests were for non-preventive purposes, within recommendations corresponds to individuals who have undergone any cancer screening (or any colorectal cancer screening) only within recommendations, while outside of recommendations corresponds to individuals who have undergone cancer screening (or any colorectal cancer screening) which includes at least one last screening test which was outside of recommendations. For FOBT, colonoscopy, mammography, uterine smear, and PSA or rectal exam, not screened corresponds to individuals who have never undergone the corresponding cancer screening test or individuals whose most recent corresponding test was for non-preventive purposes, within recommendations corresponds to individuals who have undergone the corresponding cancer screening modality and their last test was within recommendations, while outside of recommendations corresponds to individuals who have undergone the corresponding cancer screening modality and their last test was outside of recommendations.

# Table S2 – Estimated proportion of participants misclassified as having been screened within USPSTF A, B, and C recommendations (unweighted proportions)

|  | **Colorectal cancer screening (recommended to adults 45-75y/o)** | | **Breast cancer screening (recommended to women 50-74y/o)** | **Cervical cancer screening (recommended to women 21-65y/o)** | **Prostate cancer screening (none recommended)** |
| --- | --- | --- | --- | --- | --- |
| **Age group (years)** | **FOBT** | **Colonoscopy** | **Mammography** | **Uterine smear** | **PSA or rectal exam** |
| 75+ | 0.0% | 0.0% | 6.6% | 18.7% | 3.3% |
| 85+ | 0.0% | 0.0% | 22.2% | 27.0% | 6.4% |
| 80-84 | 0.0% | 0.0% | 10.5% | 19.0% | 3.5% |
| 75-79 | 0.0% | 0.0% | 0.0% | 15.5% | 2.4% |
| 70-74 | 0.0% | 0.0% | 0.0% | 6.0% | 0.0% |
| 65-69 | 0.0% | 0.0% | 0.0% | 0.0% | 0.0% |
| 60-64 | 0.0% | 0.0% | 0.0% | 0.0% | 0.0% |

Abbreviations: FOBT, fecal occult blood test; PSA, prostate-specific antigen test.

# Table S3 – Estimated proportion of participants misclassified as having been screened within USPSTF A and B recommendations (unweighted proportions)

|  | **Colorectal cancer screening (recommended to adults 45-75y/o)** | | **Breast cancer screening (recommended to women 50-74y/o)** | **Cervical cancer screening (recommended to women 21-65y/o)** | **Prostate cancer screening (none recommended)** |
| --- | --- | --- | --- | --- | --- |
| **Age group (years)** | **FOBT** | **Colonoscopy** | **Mammography** | **Uterine smear** | **PSA or rectal exam** |
| 75+ | 1.1% | 0.2% | 6.6% | 18.7% | 0.0% |
| 85+ | 6.5% | 2.3% | 22.2% | 27.0% | 0.0% |
| 80-84 | 1.7% | 0.0% | 10.5% | 19.0% | 0.0% |
| 75-79 | 0.0% | 0.0% | 0.0% | 15.5% | 0.0% |
| 70-74 | 0.0% | 0.0% | 0.0% | 6.0% | 0.0% |
| 65-69 | 0.0% | 0.0% | 0.0% | 0.0% | 0.0% |
| 60-64 | 0.0% | 0.0% | 0.0% | 0.0% | 0.0% |

Abbreviations: FOBT, fecal occult blood test; PSA, prostate-specific antigen test.

# Table S4 – Proportions of cancer screening use according to USPSTF recommendations among older adults 75+ years of age (unweighted proportions corrected for estimated age at screening misclassification)

|  | **Colorectal cancer screening (men and women)** | | **Breast cancer screening (women)** | **Cervical cancer screening (women)** | **Prostate cancer screening (men)** |
| --- | --- | --- | --- | --- | --- |
| **Screening engagement according to recommendations** | **FOBT** | **Colonoscopy** | **Mammography** | **Uterine smear** | **PSA or rectal exam** |
| Not screened  Within A, B, and C recommendations  Outside of A, B, and C recommendations | 75.2%  24.1%  0.8% | 61.2%  38.4%  0.4% | 32.7%  41.9%  25.5% | 25.4%  16.0%  58.6% | 56.7%  3.6%  39.7% |
| Not screened  Within A and B recommendations  Outside of A and B recommendations | 75.2%  14.7%  10.2% | 61.2%  28.5%  10.4% | 32.7%  41.9%  25.5% | 25.4%  16.0%  58.6% | 56.7%  0.0%  43.3% |

Abbreviations: FOBT, fecal occult blood test; PSA, prostate-specific antigen test. For FOBT, colonoscopy, mammography, uterine smear, and PSA or rectal exam, not screened corresponds to individuals who have never undergone the corresponding cancer screening test or individuals whose most recent corresponding test was for non-preventive purposes, within recommendations corresponds to individuals who have undergone the corresponding cancer screening modality and their last test was within recommendations, while outside of recommendations corresponds to individuals who have undergone the corresponding cancer screening modality and their last test was outside of recommendations.

# Table S5 – Proportions of cancer screening use according to USPSTF A, B, and C recommendations (unweighted proportions corrected for estimated age at screening misclassification)

|  | **Colorectal cancer screening (recommended to adults 45-75y/o)** | | **Breast cancer screening (recommended to women 50-74y/o)** | **Cervical cancer screening (recommended to women 21-65y/o)** | **Prostate cancer screening (none recommended)** |
| --- | --- | --- | --- | --- | --- |
| **Age group (years) and screening engagement according to recommendations** | **FOBT** | **Colonoscopy** | **Mammography** | **Uterine smear** | **PSA or rectal exam** |
| 85+  Not screened  Within recommendations  Outside of recommendations | 75.8%  19.8%  4.4% | 68.2%  29.4%  2.4% | 43.8%  18.6%  37.6% | 39.3%  15.7%  45.0% | 63.1%  2.7%  34.2% |
| 80-84  Not screened  Within recommendations  Outside of recommendations | 78.6%  21.4%  0.0% | 64.8%  35.2%  0.0% | 31.9%  30.5%  37.5% | 24.2%  17.2%  58.6% | 57.1%  3.6%  39.3% |
| 75-79  Not screened  Within recommendations  Outside of recommendations | 72.8%  27.2%  0.0% | 56.4%  43.6%  0.0% | 28.9%  55.9%  13.3% | 20.7%  15.5%  63.8% | 54.3%  3.7%  42.0% |
| 70-74  Not screened  Within recommendations  Outside of recommendations | 72.6%  27.4%  0.0% | 53.6%  46.4%  0.0% | 24.9%  75.1%  0.0% | 13.9%  20.7%  65.5% | 46.6%  23.9%  29.5% |
| 65-69  Not screened  Within recommendations  Outside of recommendations | 73.1%  26.9%  0.0% | 55.2%  44.8%  0.0% | 23.9%  76.1%  0.0% | 10.7%  60.7%  28.7% | 46.2%  53.8%  0.0% |
| 60-64  Not screened  Within recommendations  Outside of recommendations | 74.8%  25.2%  0.0% | 58.0%  42.0%  0.0% | 27.8%  72.2%  0.0% | 8.3%  91.7%  0.0% | 46.7%  53.3%  0.0% |

Abbreviations: FOBT, fecal occult blood test; PSA, prostate-specific antigen test. For FOBT, colonoscopy, mammography, uterine smear, and PSA or rectal exam, not screened corresponds to individuals who have never undergone the corresponding cancer screening test or individuals whose most recent corresponding test was for non-preventive purposes, within recommendations corresponds to individuals who have undergone the corresponding cancer screening modality and their last test was within recommendations, while outside of recommendations corresponds to individuals who have undergone the corresponding cancer screening modality and their last test was outside of recommendations.

# Table S6 – Proportions of cancer screening use according to USPSTF A and B recommendations (unweighted proportions corrected for estimated misclassification error)

|  | **Colorectal cancer screening (recommended to adults 45-85y/o** | | **Breast cancer screening (recommended to women 40-74y/o)** | **Cervical cancer screening (recommended to women 21-65y/o)** | **Prostate cancer screening (recommended to men 55-69y/o)** |
| --- | --- | --- | --- | --- | --- |
| **Age group (years) and screening engagement according to recommendations** | **FOBT** | **Colonoscopy** | **Mammography** | **Uterine smear** | **PSA or rectal exam** |
| 85+  Not screened  Within recommendations  Outside of recommendations | 75.8%  6.4%  17.8% | 68.2%  9.6%  22.3% | 43.8%  18.6%  37.6% | 39.3%  15.7%  45.0% | 63.1%  0.0%  36.9% |
| 80-84  Not screened  Within recommendations  Outside of recommendations | 78.6%  7.4%  13.9% | 64.8%  21.0%  14.2% | 31.9%  30.5%  37.5% | 24.2%  17.2%  58.6% | 57.1%  0.0%  42.9% |
| 75-79  Not screened  Within recommendations  Outside of recommendations | 72.8%  21.0%  6.3% | 56.4%  39.5%  4.1% | 28.9%  55.9%  13.3% | 20.7%  15.5%  63.8% | 54.3%  0.0%  45.7% |
| 70-74  Not screened  Within recommendations  Outside of recommendations | 72.6%  27.4%  0.0% | 53.6%  46.4%  0.0% | 24.9%  75.1%  0.0% | 13.9%  20.7%  65.5% | 46.6%  0.05%  53.4% |
| 65-69  Not screened  Within recommendations  Outside of recommendations | 73.1%  26.9%  0.0% | 55.2%  44.8%  0.0% | 23.9%  76.1%  0.0% | 10.7%  60.7%  28.7% | 46.2%  0.0%  53.8% |
| 60-64  Not screened  Within recommendations  Outside of recommendations | 74.8%  25.2%  0.0% | 58.0%  42.0%  0.0% | 27.8%  72.2%  0.0% | 8.3%  91.7%  0.0% | 46.7%  0.0%  53.3% |

Abbreviations: FOBT, fecal occult blood test; PSA, prostate-specific antigen test. For FOBT, colonoscopy, mammography, uterine smear, and PSA or rectal exam, not screened corresponds to individuals who have never undergone the corresponding cancer screening test or individuals whose most recent corresponding test was for non-preventive purposes, within recommendations corresponds to individuals who have undergone the corresponding cancer screening modality and their last test was within recommendations, while outside of recommendations corresponds to individuals who have undergone the corresponding cancer screening modality and their last test was outside of recommendations.

# Figure S4 – Visual overview of cancer screening use according to USPSTF A, B, and C recommendations with estimate of misclassification error (unweighted proportions)


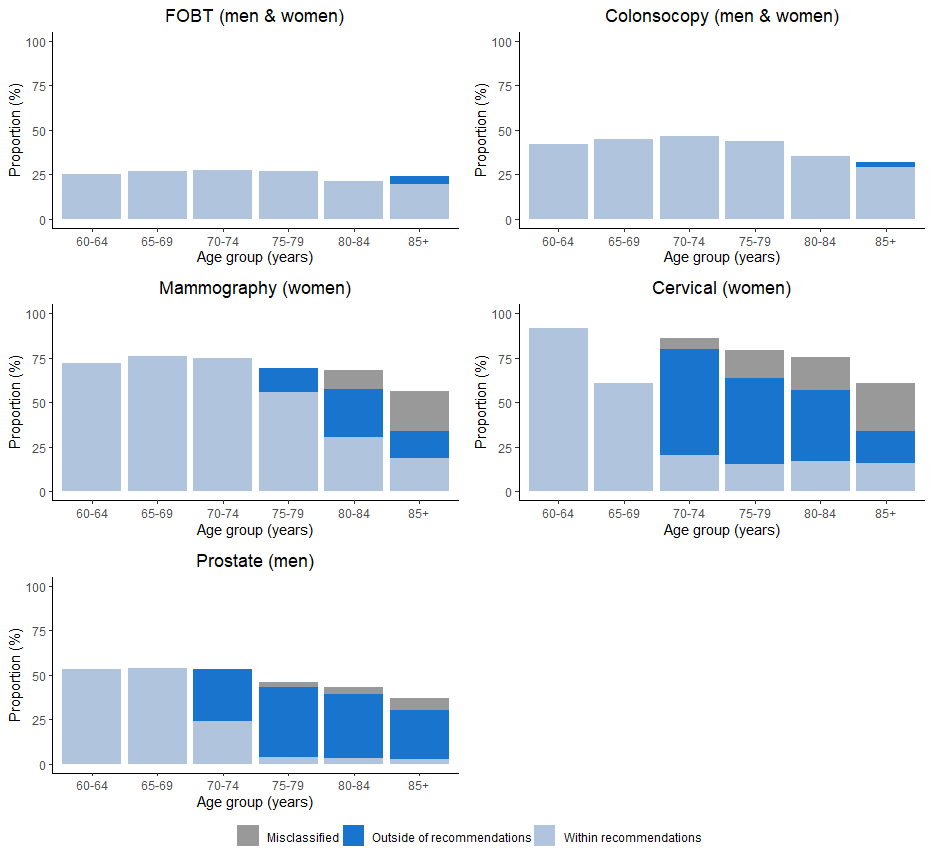


Abbreviations: FOBT, fecal occult blood test; PSA, prostate-specific antigen test. Within recommendations corresponds to individuals who have undergone the corresponding cancer screening modality and their last test was within recommendations, outside of recommendations corresponds to individuals who have undergone the corresponding cancer screening modality and their last test was outside of recommendations, and misclassified corresponds to the estimated proportion of individuals who were misclassified as having been screened within recommendations instead of having been screened outside of recommendations.

# Figure S5 – Visual overview of cancer screening use according to USPSTF A and B recommendations with estimate of misclassification error (unweighted proportions)


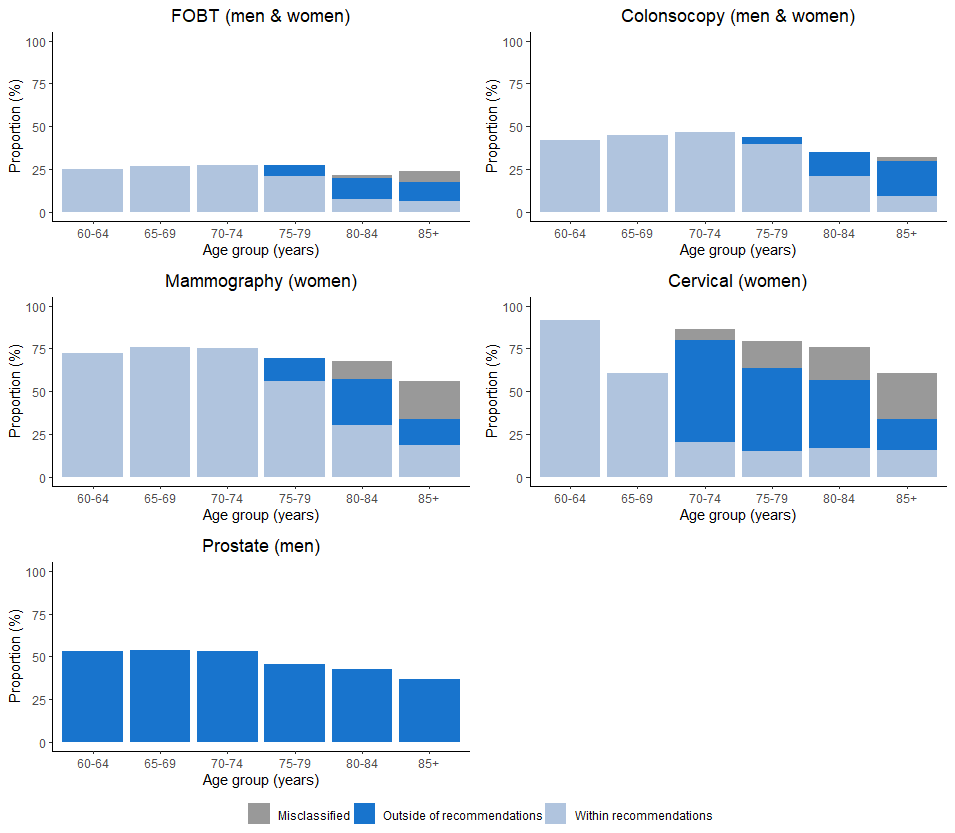


Abbreviations: FOBT, fecal occult blood test; PSA, prostate-specific antigen test. Within recommendations corresponds to individuals who have undergone the corresponding cancer screening modality and their last test was within recommendations, outside of recommendations corresponds to individuals who have undergone the corresponding cancer screening modality and their last test was outside of recommendations, and misclassified corresponds to the estimated proportion of individuals who were misclassified as having been screened within recommendations instead of having been screened outside of recommendations.

# Table S7 – Strengthening the reporting of observational studies in epidemiology (STROBE) checklist

|  | Item No | Recommendation | Location |
| --- | --- | --- | --- |
| **Title and abstract** | 1 | (*a*) Indicate the study’s design with a commonly used term in the title or the abstract | Abstract |
|  |  | (*b*) Provide in the abstract an informative and balanced summary of what was done and what was found | Abstract |
| Introduction | | |  |
| Background/rationale | 2 | Explain the scientific background and rationale for the investigation being reported | Introduction |
| Objectives | 3 | State specific objectives, including any prespecified hypotheses | Abstract; Introduction |
| Methods | | |  |
| Study design | 4 | Present key elements of study design early in the paper | Data source |
| Setting | 5 | Describe the setting, locations, and relevant dates, including periods of recruitment, exposure, follow-up, and data collection | Data source; Population |
| Participants | 6 | (*a*) Give the eligibility criteria, and the sources and methods of selection of participants | Population |
| Variables | 7 | Clearly define all outcomes, exposures, predictors, potential confounders, and effect modifiers. Give diagnostic criteria, if applicable | Variables of interest; Participant characteristics variables (supp.) |
| Data sources/ measurement | 8* | For each variable of interest, give sources of data and details of methods of assessment (measurement). Describe comparability of assessment methods if there is more than one group | Variables of interest; Participant characteristics variables (supp.) |
| Bias | 9 | Describe any efforts to address potential sources of bias | Statistical analysis; Estimating misclassification of age at screening |
| Study size | 10 | Explain how the study size was arrived at | Data source; Population |
| Quantitative variables | 11 | Explain how quantitative variables were handled in the analyses. If applicable, describe which groupings were chosen and why | Variables of interest; Participant characteristics variables (supp.); Statistical analysis |
| Statistical methods | 12 | (*a*) Describe all statistical methods, including those used to control for confounding | Statistical analysis |
|  |  | (*b*) Describe any methods used to examine subgroups and interactions | Statistical analysis |
|  |  | (*c*) Explain how missing data were addressed | Population |
|  |  | (*d*) If applicable, describe analytical methods taking account of sampling strategy | Statistical analysis |
|  |  | (*e*) Describe any sensitivity analyses | Estimating misclassification of age at screening |
| Results | | |  |
| Participants | 13* | (a) Report numbers of individuals at each stage of study—eg numbers potentially eligible, examined for eligibility, confirmed eligible, included in the study, completing follow-up, and analysed | Data source; Population; Figure S1 |
|  |  | (b) Give reasons for non-participation at each stage | Figure S1 |
|  |  | (c) Consider use of a flow diagram | Figure S1 |
| Descriptive data | 14* | (a) Give characteristics of study participants (eg demographic, clinical, social) and information on exposures and potential confounders | Table 1 |
|  |  | (b) Indicate number of participants with missing data for each variable of interest | Table 1 |
| Outcome data | 15* | Report numbers of outcome events or summary measures | Tables 2, 3, S1 |
| Main results | 16 | (*a*) Give unadjusted estimates and, if applicable, confounder-adjusted estimates and their precision (eg, 95% confidence interval). Make clear which confounders were adjusted for and why they were included | N/A |
|  |  | (*b*) Report category boundaries when continuous variables were categorized | N/A |
|  |  | (*c*) If relevant, consider translating estimates of relative risk into absolute risk for a meaningful time period | N/A |
| Other analyses | 17 | Report other analyses done—eg analyses of subgroups and interactions, and sensitivity analyses | Estimated misclassification of age at screening |
| Discussion | | |  |
| Key results | 18 | Summarise key results with reference to study objectives | Discussion |
| Limitations | 19 | Discuss limitations of the study, taking into account sources of potential bias or imprecision. Discuss both direction and magnitude of any potential bias | Limitations |
| Interpretation | 20 | Give a cautious overall interpretation of results considering objectives, limitations, multiplicity of analyses, results from similar studies, and other relevant evidence | Discussion |
| Generalisability | 21 | Discuss the generalisability (external validity) of the study results | Limitations |
| Other information | | |  |
| Funding | 22 | Give the source of funding and the role of the funders for the present study and, if applicable, for the original study on which the present article is based | Footnotes (Funding) |
